# Changes in Season Affect Body Weight, Physical Activity, Food Intake, and Sleep in Female College Students: A Preliminary Study

**DOI:** 10.3390/ijerph17238713

**Published:** 2020-11-24

**Authors:** Eiichi Yoshimura, Eri Tajiri, Yoichi Hatamoto, Shigeho Tanaka

**Affiliations:** 1 Department of Nutrition and Metabolism, National Institute of Health and Nutrition, National Institutes of Biomedical Innovation, Health and Nutrition, Tokyo 162-8636, Japan; yhatamoto@nibiohn.go.jp (Y.H.); tanakas@nibiohn.go.jp (S.T.); 2 Graduate School of Environmental & Symbiotic Sciences, Prefectural University of Kumamoto, 3-1-100 Tsukide, Higashi-ku, Kumamoto 862-8502, Japan; g1975002@pu-kumamoto.ac.jp

**Keywords:** weight gain, seasonal variability, physical activity, college students

## Abstract

The current study examined how body weight and lifestyle fluctuate between spring, autumn, and winter in Japanese female college students and whether weight gain is associated with changes in physical activity, food intake, and sleep. We measured body weight and lifestyle factors in 31 participants from May 2017 to January 2018. Weight was measured daily in participants’ homes. Physical activity and sleep were measured for three weeks in three seasons using two accelerometers. Food intake was assessed using a validated food frequency questionnaire. Body weight significantly decreased in autumn compared with spring (*p* < 0.001). Body weight in winter tended to increase compared with autumn (*p* = 0.052). Step counts and energy intake were significantly different between seasons (*p* < 0.05). Total time in bed was not significantly different between seasons. In comparisons of changes in lifestyle patterns from autumn to winter between the weight gain (≥0.5 kg) and weight maintenance groups, seasonal changes in lifestyle factors were not significantly different between groups (*p* > 0.05). The results indicated that body weight and lifestyle were affected by seasonal variability in female college students, but no significant relationships existed between seasonal weight gain and changes in lifestyle patterns.

## 1. Introduction

Since 1980, the prevalence of obesity has doubled in more than 70 countries [1]. Globally, 603.7 million adults were estimated to be obese in 2015 [1]. According to the 2017 National Health and Nutrition Survey in Japan, the average body mass index (BMI) score among young Japanese women was very low compared with many other countries (15–19-years age group: 20.7 kg/m^2^ and 20–29-years age group: 20.6 kg/m^2^). Only 6.4% of women aged 15–19 years and 5.7% of women aged 20–29 years in Japan were classified as overweight (BMI ≥ 25 kg/m^2^), respectively. However, this proportion was reported to increase with age, and one in four women in Japan aged ≥60 years was found to be overweight, and women ≥60 years old were approximately four times more likely to be overweight than those aged 20–29 years [2].

Thus, this increase in body weight with age might be affected by changes in the social environment, such as changes in employment, marriage, and family size with increasing age. College students experience drastic changes in living environments compared with high school students, and body weights tend to increase during this period [3,4]. Gropper et al. reported that college students gained approximately 3.0 kg on average over the four-year college period [4]. In addition, approximately 70% of participants gained body weight [4].

Body weight also varies by season. A recent study reported that Japanese adults exhibit an average weight increase of approximately 0.5% in the holiday season, including Christmas and New Year’s [5]. During the Golden Week holidays in Japan in May, body weights were reported to be higher than any other time of year [5]. Yanovski et al. reported a 0.48-kg increase in body weight between autumn and winter in adults, causing increased research attention on holiday weight gain [6]. Since individuals commonly exhibit weight gain during college, weight management over the college-aged period is considered important for lifetime body weight management. Hull et al. reported that college students gained a significant amount of body weight (0.5 kg) during the Thanksgiving holiday [7]. However, a recent review emphasized that relatively few studies have examined college students [8]. Thus, although some studies have evaluated changes in body weight during the four-year college period, few studies have assessed holiday or seasonal changes in body weight among college students. In addition, although body weight varies from day to day, many studies only evaluated body weight once per season. Since this measurement method cannot take the effects of daily variations in body weight into account, the assessment of seasonal variations in body weight requires the averaging of continuous data. Thus, the details of holiday and seasonal weight variations in college students remain to be elucidated.

It is possible that individuals who gain weight during holidays or seasons exhibit specific characteristics, such as decreased physical activity, increased food intake, and sleep curtailment. However, to our knowledge, no previous studies have assessed holiday or seasonal changes in body weights, as well as examining lifestyle habits, in college students. Thus, it is currently unclear which lifestyle factors influence weight gain during the holiday period.

The purpose of the current study was to clarify how body weight, assessed using continuous data, fluctuates from spring to winter (May to January) among Japanese college students and to clarify whether weight gain is associated with changes in lifestyle factors, such as physical activity, food intake, and sleep.

## 2. Methods

### 2.1. Participants

We recruited college students according to the following inclusion criteria: (1) healthy women without diabetes, hypertension, or dyslipidemia; (2) college students who did not have part-time jobs starting at midnight; (3) nonsmoking; (4) no habit of regular exercise; and (5) first- to fourth-year college students. Thirty-three college students aged 21.6 ± 0.5 years (age range: 18–22 years) who met these criteria took part in the study. Participants included eight first-year students, 11 second-year students, 12 third-year students, and two fourth-year students. Two participants were excluded from the analysis because of diagnoses of depression and hypothyroidism during the study period. Participants were instructed to measure their weight and lifestyle factors, including physical activity, food intake, and sleep, between May 2017 and January 2018. Participants were instructed to measure their weight daily in their own homes as consistently as possible over this time. Physical activity and sleep were assessed for 3 weeks in the spring, autumn, and winter. Data corresponded to each Japanese season between the following dates: spring, 14 May to 27 June, autumn, 2 October to 1 November, and winter, 1 January to 31 January. Food intake was assessed at the same time using a validated food frequency questionnaire. The study protocol was explained, and written informed consent was obtained from all participants. The study protocol was approved by the Ethics Committee of the Prefectural University of Kumamoto (No. 29-16).

### 2.2. Anthropometric Measurement

For measurements of body weight, participants were provided with a body composition meter (BC-308, TANITA, Tokyo, Japan) that was able to conduct measurements in units of 50 g. Participants were instructed to weigh themselves daily under fasting conditions within 1 h of waking in the morning, while wearing clothing that was as similar as possible, to minimize the effects on weight measurements. Participants were instructed that, when they were traveling or attending training, it was not necessary to take weight measurements. Body weight and time data were stored on a built-in SD card. Height was measured to the nearest 0.1 cm using digital scales with a stadiometer. BMI scores were calculated as kg/m^2^.

### 2.3. Physical Activity

Physical activity was measured for 3 weeks in 1-min epochs using a tri-axial accelerometer (HJA-750C; Omron Healthcare, Kyoto, Japan) [9] in spring, autumn, and winter. Participants wore the accelerometer on their waist throughout the measurement period, except while sleeping or bathing. Physical activity was assessed using step count and activity time by intensity levels. The obtained physical activity intensity level in each minute were classified into three categories (sedentary behavior, ≤1.5 metabolic equivalents (METs), light, 1.6 to 2.9 METs, and moderate-to-vigorous, ≥3.0 METs). The amount of time spent at each intensity level was summed across a day. Nonwear time was defined as a consecutive zero count of 60 min or more. Accelerometer data were adopted if there was more than 600 min of monitoring per day. Percentage of activity time by intensity levels of worn time was used to examine differences in seasonal variability.

### 2.4. Sleep

Into-bed time, wake-up time, total time in bed, total sleep time, and sleep efficiency were assessed using an MTN-220 (ACOS, Nagano, Japan) device worn at the waist and compared with sleep logs to validate sleep time. All devices were configured to record activity every 2 min. A 2-min epoch was used, because this time window is often used to save memory space when long-term activity logging is required. For sleep/wake detection from MTN-220 data, default settings in the SleepSign Act were used, in which sleep detection followed a previously reported algorithm [10]. The MTN-220 was worn before bedtime and removed after wake-up time. These measurements were conducted for 3 weeks in spring, autumn, and winter seasons.

### 2.5. Dietary Intake

Food intake was assessed using a validated food frequency questionnaire (FFQg: Excel Eiyo-kun FFQg, version 4.0, Kenpaku-sha, Tokyo, Japan) [11]. The FFQg is composed of items in 29 food groups and 10 kinds of cookery. Participants reported the foods they consumed in the past 1 to 2 months by selecting a portion size and frequency of each food group and the average intake per week. After participants completed the questionnaire, a dietician checked the completed questionnaire with the participant.

### 2.6. Statistical Analysis

Bonferroni’s correction was used for multiple comparisons of body weight, physical activity, energy intake, and sleep conditions for three seasons. Student’s unpaired *t*-tests were used to compare body weight, physical activity, energy intake, and sleep conditions between the weight gain and maintenance groups. For assessment of seasonal variations in body weight, daily body weight in each participant was smoothed over a 7-day running-average window and averaged over the participants in groups. Based on previous studies [6,12], participants whose body weights increased by ≥0.5 kg from autumn to winter were included in the weight gain group. Participants whose weights did not increase by ≥0.5 kg were included in the weight maintenance group. All data are presented as means ± standard deviations. The significance level for all statistical tests was set at *p* < 0.05. All data were analyzed using IBM SPSS Statistics for Windows 8.1 (version 22.0; IBM Corp, Armonk, NY, USA).

## 3. Results

In spring, the mean body weight was 52.7 ± 4.7 kg and the mean BMI score was 21.1 ± 1.6 kg/m^2^. During the measurement period, daily weight measurements were available for 31 participants, which contributed a total of 5871 days or 838 weeks of data. Regarding compliance with the assessment items during the measurement period, the measurement rate during the three seasons was 88.0 ± 9.0% for body weight, 90.9 ± 7.9% for physical activity, and 80.4 ± 13.7% for sleep condition.

### 3.1. Daily Body Weight

**Figure 1:**
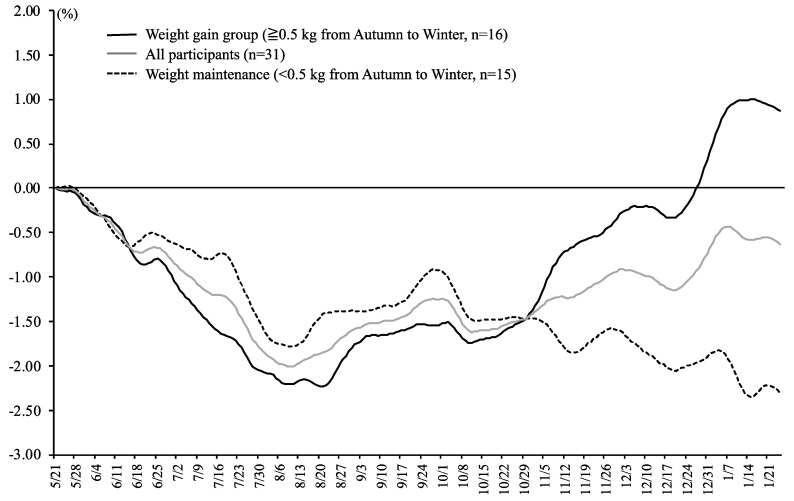
Changes in daily body weight from May 2017 to January 2018. For assessment of the seasonal variations in body weight, daily body weight in each participant was smoothed over a 7-day running-average window and averaged across participants in groups. Based on previous studies, participants in which body weight increased by ≥0.5 kg from autumn to winter were included in the weight gain group, while participants who did not show weight increases of ≥0.5 kg were included in the weight maintenance group.

Table 1 shows seasonal variability in body weight and lifestyle among female college students. Body weight significantly decreased in autumn compared with spring (−0.86 ± 1.18 kg, *p* < 0.001). Body weight in winter increased compared with autumn, although the difference did not reach significance (0.52 ± 1.15 kg, *p* = 0.052). The results of body weight variability (SD) and the range in body weight during the measurement period was similar between seasons. The changes in daily body weight from May 2017 to January 2018 are shown in Figure 1. Participants’ body weight tended to decrease from the beginning of the study to the summer season, and tended to increase from summer to winter.

### 3.2. Food Intake

Energy intake significantly decreased in winter compared with autumn (−134 ± 221 kcal/day, *p* = 0.006). The percentage of protein (−1.4 ± 2.2%, *p* = 0.003) and fat intake (−3.2 ± 5.5%, *p* = 0.003) significantly decreased in winter compared with autumn.

### 3.3. Physical Activity

Step counts significantly decreased in the winter season compared with spring (−1340 ± 1542 steps, *p* < 0.001) and autumn (−854 ± 1513 steps, *p* = 0.011). The percentage of moderate-to-vigorous intensity of physical activity also significantly decreased in winter compared with spring (−1.0 ± 1.5%, *p* < 0.001) and autumn (−1.6 ± 1.8%, *p* = 0.002).

### 3.4. Sleep

Total time in bed was not significantly different during the three seasons studied. Into-bed time was delayed in winter compared with spring (36 ± 38 min, *p* = 0.005) and autumn (49 ± 42 min, *p* = 0.021). Wake-up time was also delayed in winter compared with spring (42 ± 68 min, *p* < 0.001) and autumn (26 ± 61 min, *p* < 0.001). The details of these results are shown in Table 1.

### 3.5. Changes of Lifestyle Patterns in Weight Gain and Maintenance Groups

Changes in lifestyle patterns from autumn to winter between the weight gain and weight maintenance groups are shown in Table 2. Body weight in the weight gain group increased from autumn to winter compared with the weight maintenance group (1.46 ± 0.56 kg vs. −0.50 ± 0.59 kg, *p* < 0.001). This result did not change after adjusting for the effects of BMI or body weight at the start of the study. A comparison of the seasonal changes between groups revealed no significant relationships between seasonal weight gain and changes in lifestyle patterns, such as physical activity, food intake, and sleep conditions.

## 4. Discussion

The present study examined seasonal variations in body weight, physical activity, food intake, and sleep conditions among female college students. The results indicated that body weight, physical activity, food intake, and sleep conditions were affected by season. Seasonal changes in body weight assessed from continuous data (a total of 5871 days or 838 weeks of data) were similar to those reported in previous studies [5]; body weight tended to decrease from spring to summer and tended to increase from summer to winter. In winter, step counts and moderate-to-vigorous intensity physical activity decreased, and the duration of sedentary behavior increased. For variability in sleep conditions, the total time in bed did not significantly change between spring, autumn, and winter, but into-bed time and wake-up time were delayed in winter. Changes in lifestyle factors, such as physical activity, food intake, and sleep conditions, were not associated with weight gain. To the best of our knowledge, no previous studies have assessed holiday or seasonal changes in body weight together with lifestyle habits in college students. Thus, this preliminary study is the first report assessing the relationship between lifestyle factors and weight gain during the holiday period.

A recent review indicated that body weight during the holiday season (defined as starting from the last week of November to the first or second week of January) increased by approximately 0.4 to 0.9 kg [8]. Another study reported that many adults in the United States exhibit annual weight gains of 0.5 to 1.0 kg [13]. Other studies have reported that weight gain through the holiday season, including Thanksgiving, Christmas, and New Year’s, may account for most of the weight gain in a year [6]. In the current study, we hypothesized that female college students in Japan would also exhibit an increase in body weight in the winter. However, no obvious peak was observed. Compared with autumn, the change in body weight measured after New Year’s revealed that the difference was not large (*p* = 0.052). In addition, body weight in the second week of January was also no different compared with the last week of November (0.30 ± 1.00 kg, *p* = 0.401). Hull et al. examined college students, reporting no difference between the initial pre-Thanksgiving weight and final post-New Year’s weight (71.3 kg vs. 71.2 kg; difference: −0.1 kg, *p* = 0.710) [14]. The current findings are consistent with the results of Hull et al. [14]. However, in another study by Hull et al., it was reported that college students gained a significant amount of body weight (0.5 kg) between pre- and post-Thanksgiving [7]. The timing of the evaluation period may have affected the results. In addition, the holiday season differs between countries. Helander et al. reported that individuals in Germany and the United States tended to gain weight from early November, while individuals in Japan did not exhibit body weight increases until mid-December [5]. Japanese individuals have been reported to specifically gain weight during the Golden Week holidays. The current results indicate that seasonal variability in body weight occurred among female college students in Japan. Differences in body weight (maximum–minimum) during the measurement period were 2.40 ± 0.72 kg (4.6 ± 1.4%), indicating substantial variability.

The current findings indicated that physical activity decreased in winter compared with spring and autumn. Physical activity may also be affected by a variety of environmental factors, such as temperature and precipitation [15]. However, Wang et al. reported that there was no seasonal variation in physical activity and no significant impact of temperature in 40 Han Chinese adults living in Beijing [16]. Consistent with the current results, previous studies reported that the lowest levels of physical activity were observed in the winter, with the highest levels in spring [17]. Thus, cold temperatures in the winter could potentially reduce the amount of physical activity. Decreased physical activity in the winter might lead to weight gain, but changes in physical activity were no different between the weight gain group (≥0.5 kg from autumn to winter) and the weight maintenance group in the present study. Cook et al. investigated whether physical activity, assessed using the doubly labeled water method, has a protective effect on weight gain from autumn to winter [18]. The results revealed that physical activity at the baseline was not significantly associated with weight gain. Stevenson et al. reported that regular, self-reported exercise of more than 150 min/week at moderate-to-vigorous intensity was not a predictor for weight change after the holiday season [19]. Since no previous studies have examined the effects of seasonal weight gain and physical activity among college students, additional studies are needed.

Although the current study indicated that the total time spent in bed was not significantly different between the three seasons, the into-bed time and wake-up time were delayed in winter. In addition, the total sleep time and sleep efficiency were significantly lower in autumn compared with spring and winter. In contrast, Lehnkering et al. reported that the sleep-time duration monitored by the Actiwatch accelerometer was longer in autumn (November) than spring (May) among college students [20]. Since the timing of the evaluation period differed between this previous study and the current study, a simple comparison is not possible. However, there appears to be a difference in sleep time depending on the season. In addition, features of the school schedule, such as vacations and regularly scheduled examinations, may also cause biases. The current results revealed no significant differences in sleep conditions between the weight gain group and the weight maintenance group. However, Ludy et al. reported that first-year college students who gained body weight during the beginning of the fall (August) and spring semesters (January) exhibited decreased sleep time compared with students who maintained the same weight [21]. This difference in results may be due to differences in habitual sleep times. It is well-known that the average sleep time in Japan is relatively short compared with that in many other countries [22]. Participants in the current study exhibited a total sleep time of approximately 5.5 to 6.0 h, whereas the total sleep time reported by Ludy et al. was approximately 6.7 to 7.6 h [21]. In addition, the differences may have also been related to the smaller changes in body weight during the holiday period in the current study compared with the previous study (0.52 kg vs. 1.8 kg).

In the current preliminary study, energy intake, as assessed by the FFQ, differed between seasons and significantly decreased in the winter compared with autumn. The winter involves many holiday events, such as Christmas and New Year’s, and is expected to involve a variety in meal contents [23], high-energy density meals [24], and large portion sizes [25]. Participants reported the foods they consumed in the past one to two months by selecting the portion size and frequency of each food group. Although a dietician checked the completed questionnaire with each participant, recall bias may have affected the results [26]. However, because participants did not gain weight from the autumn to winter, they may not have actually increased their energy intake. No associations between seasonal change, diet score, and body weight among college students were reported in a previous study [21]. Further studies are needed to examine this issue using food record methods.

A recent review reported that there is a lack of studies investigating the mediators of weight gain during the holiday season [8]. Thus, we sought to clarify whether weight gain among female college students was associated with changes in lifestyle factors, such as physical activity, food intake, and sleep. However, regarding changes in lifestyle patterns from the autumn to winter between weight gain and weight maintenance groups, the results revealed no significant relationships between weight variability and lifestyle patterns. Interestingly, even over the holiday period, which involves lifestyle changes, only approximately half of the participants gained 0.5 kg or more in body weight, compared with the autumn. Thus, half of the participants did not gain weight during the winter. In addition, compared with the spring, only six participants gained 0.5 kg or more of body weight in the winter. There are long vacations three times per year (summer, winter, and spring) for Japanese college students, and the summer and spring vacations are approximately 1.5 months long. Thus, weight variations during long vacations among college students might affect weight control more than the winter holiday season. Additional studies will be needed to assess weight variability throughout the whole year.

The current study involved several potential limitations that should be considered. First, the study had a relatively small sample size. It will be necessary to confirm the current results with a larger sample. Next, we were only able to recruit two fourth-year college students, because students at this stage of study are occupied by job hunting. In addition, because all participants in this study belonged to the same university and department, the results may not be representative of all female college students. Ma et al. reported that the education level of participants can affect seasonal variations in food intake, physical activity, and body weight [27]. While approximately 60% of high school students in Japan go on to college, it is also necessary to research this issue among noncollege students. Although we instructed participants not to change their usual lifestyles during the survey period in the current study, it is possible that participants’ lifestyles changed as a result of the weight measurements. Such changes in lifestyle rhythms can be difficult to detect. Thus, it may be helpful for future studies to test the effects of using a different measurement start dates. The current study collected data on food intakes, physical activity, sleep conditions, and body weight at the same time in three different seasonal periods, representing a strength of the study design.

## 5. Conclusions

The current results indicate that body weight, physical activity, food intakes, and sleep conditions are affected by season in female college students, but no significant relationships exist between seasonal weight gain and changes in lifestyle patterns. Individuals who gain weight are often assumed to have increased their energy intake and/or decreased their energy expenditure; however, the current findings suggested little differences in these factors. Additional research should be conducted in combination with a study design involving controlled conditions using a metabolic chamber to detect small differences.

## Figures and Tables

**Table 1:** Seasonal variability in body weight, physical activity, food intake, and sleep in female college students.

	Spring Season			Autumn Season			Winter Season			*p*-Value		
										Spring vs. Autumn	Spring vs. Winter	Autumn vs. Winter
Weight (kg)	52.7	±	4.7	51.8	±	5.1	52.3	±	5.4	**		‡
Weight SD (kg)	0.6	±	0.2	0.4	±	0.1	0.5	±	0.2	**		
The range of weight variability (Max–Min) (kg)	2.1	±	0.7	1.7	±	0.5	1.8	±	0.7	*		
Body mass index (kg/m^2^)	21.1	±	1.6	20.7	±	1.7	20.9	±	1.7	**		
Step counts (steps)	7492	±	1747	7007	±	2014	6152	±	1483		***	*
Physical inactivity (%)	61.0	±	7.5	63.0	±	7.1	65.6	±	7.1		**	
Light intensity physical activity (%)	32.6	±	6.4	31.2	±	6.0	29.7	±	6.7			
Moderate-to-vigorous intensity physical activity (%)	6.4	±	2.0	5.7	±	2.0	4.7	±	1.2		***	**
Energy intake (kcal/day)	1803	±	403	1822	±	367	1688	±	297			**
Protein (g/day)	65	±	17	66	±	16	58	±	13		*	**
Fat (g/day)	68	±	16	69	±	16	61	±	13		*	**
Carbohydrate (g/day)	224	±	56	226	±	48	217	±	44			
Protein (%)	14.4	±	1.6	14.4	±	1.3	12.9	±	2.0		**	**
Fat (%)	34.3	±	4.7	33.9	±	4.3	30.7	±	5.3		**	**
Carbohydrate (%)	49.6	±	5.6	49.7	±	4.8	48.4	±	8.0			
Cereals (rice, boiled noodles, etc.) (g/1000 kcal)	186.3	±	54.7	186.9	±	46.8	191.8	±	46.8			
Potatoes (g/1000 kcal)	15.9	±	13.4	15.3	±	11.7	17.2	±	14.8			
Green and yellow vegetables (g/1000 kcal)	41.3	±	28.1	34.3	±	14.5	35.7	±	16.9			
Other vegetables (g/1000 kcal)	64.9	±	31.1	58.7	±	26.3	62.0	±	26.3			
Seaweeds (g/1000 kcal)	1.6	±	2.0	1.5	±	1.4	1.6	±	1.8			
Beans (g/1000 kcal)	31.0	±	20.0	32.1	±	18.9	27.0	±	15.8			
Fish and seafoods (g/1000 kcal)	19.0	±	14.2	19.7	±	12.0	17.9	±	12.3			
Meats (g/1000 kcal)	57.3	±	24.7	58.0	±	16.6	55.8	±	23.6			
Eggs (g/1000 kcal)	24.1	±	9.8	21.4	±	9.9	21.4	±	7.6			
Milk and dairy products (g/1000 kcal)	93.9	±	45.0	77.1	±	46.2	81.4	±	48.8			
Fruits (g/1000 kcal)	29.4	±	33.4	25.6	±	20.2	38.7	±	41.9			
Confectioneries (g/1000 kcal)	40.9	±	17.8	44.1	±	18.1	45.2	±	22.8			
Beverages (g/1000 kcal)	29.5	±	36.6	35.1	±	50.0	25.1	±	34.2			
Sugar and sweeteners (g/1000 kcal)	3.0	±	1.9	2.7	±	1.7	2.9	±	2.3			
Nuts and seeds (g/1000 kcal)	0.9	±	1.6	0.7	±	1.0	0.7	±	1.1			
Oils and fats (g/1000 kcal)	7.7	±	3.1	7.2	±	3.3	6.4	±	2.4			
Seasonings and spices (g/1000 kcal)	12.8	±	6.7	12.6	±	6.5	13.0	±	7.2			
Into-bed time (hr:min) ^†^	24:52	±	1:03	25:04	±	0:54	25:35	±	0:54		**	*
Wake-up time (hr:min) ^†^	7:50	±	1:02	8:03	±	0:58	8:39	±	0:46		***	***
Total time in bed (min) ^†^	418	±	41	420	±	45	425	±	35			
Total sleep time (min) ^†^	358	±	46	335	±	46	366	±	51	**		**
Sleep efficiency (%) ^†^	85.7	±	6.8	79.7	±	7.1	85.8	±	7.6	***		***

Comparison in season variability was analyzed using Bonferroni’s correction. Asterisks denote statistical significance of seasonal variations and * *p* < 0.05, ** *p* < 0.01, *** *p* < 0.001. Dagger (^†^) mean *n* = 30. Double dagger (‡) mean *p* = 0.052. SD, standard deviation.

**Table 2:** Change in lifestyle patterns from autumn to the winter season between weight gain and weight maintenance groups.

	Weight Gain Group (*n* = 16)			Weight Maintenance Group (*n* = 15)			*p*-Value
	Av	±	SD	Av	±	SD	
∆Weight (kg)	1.46	±	0.56	−0.50	±	0.59	<0.001
∆Physical inactivity (min)	19	±	75	40	±	102	0.514
∆Light intensity physical activity (min)	1	±	41	−27	±	53	0.118
∆Moderate-to-vigorous intensity physical activity (min)	−9	±	15	−10	±	13	0.859
∆Accelerometer wear time (min)	10	±	80	3	±	68	0.786
∆Step counts (steps)	−942	±	1566	−760	±	1504	0.745
∆Physical inactivity (%)	1.3	±	4.5	3.9	±	7.4	0.238
∆Light intensity physical activity (%)	−0.3	±	3.9	−2.9	±	6.4	0.184
∆Moderate-to-vigorous intensity physical activity (%)	−1.0	±	1.5	−1.0	±	1.4	0.905
∆The into-bed time (h:min)	0:38	±	1:11	0:14	±	0:46	0.289
∆Wake-up time (h:min)	0:32	±	0:40	0:41	±	0:37	0.542
∆Total time in bed (min)	4	±	63	37	±	50	0.115
∆Total Sleep time (min)	22	±	57	47	±	43	0.170
∆Sleep efficiency (%)	6	±	5	6	±	4	0.933
∆Energy intake (kcal/day)	−145	±	232	−123	±	216	0.793
∆Protein (g/day)	−8.9	±	11.6	−6.0	±	8.9	0.453
∆Fat (g/day)	−8.0	±	12.3	−6.7	±	9.4	0.752
∆Carbohydrate (g/day)	−7.0	±	35.2	−9.7	±	32.3	0.825
∆Protein (%)	−1.8	±	2.5	−1.0	±	1.8	0.310
∆Fat (%)	−3.8	±	6.0	−2.6	±	5.1	0.549
∆Carbohydrate (%)	−1.4	±	7.9	−1.2	±	7.1	0.949

Data show the amount of changes between autumn and winter.
